# The prevalence of intestinal parasite infection and associated factors among food handlers in eating and drinking establishments in Chagni Town, Northwest Ethiopia

**DOI:** 10.1186/s13104-019-4338-5

**Published:** 2019-05-28

**Authors:** Aschale Shimeles Alemu, Adhanom Gebreegziabher Baraki, Mekuriaw Alemayehu, Melaku Kindie Yenit

**Affiliations:** 1 Chagni Health Office, Awi Zone, Chagni, Ethiopia; 2 Department of Epidemiology and Biostatistics, Institute of Public Health, University of Gondar, Gondar, Ethiopia; 3 Department of Environmental and Occupational Safety, Institute of Public Health, University of Gondar, Gondar, Ethiopia

**Keywords:** Prevalence, Intestinal parasites, Food handlers, Food and drink establishment, Chagni Town, Ethiopia

## Abstract

**Objectives:**

This study aimed to determine the prevalence of intestinal parasites and associated factors among food handlers in Changni district, Awi zone Ethiopia.

**Results:**

A total of 442 food handlers were included in the study with a response rate of 90%. The prevalence of infection with at least one of the intestinal parasites was 14.8% [95% CI (11.5%, 18.0%)]. According to multivariable logistic regression analysis, lack of regular hand washing before meal [AOR = 4.77, 95% CI (2.09, 10.87)], regular hand washing after visiting toilets, [AOR = 3.39, 95% CI (1.52, 7.57)], trimmed fingernails, [AOR = 2.39, 95% CI (1.29, 4.42)], and frequent medical check-ups for intestinal parasites [AOR = 3.54, 95% CI (1.11, 11.31)] were significantly associated with the infection of intestinal parasitosis.

## Introduction

Parasitic infections remain one of the major public health problems in areas where there is poor personal hygiene and environmental sanitation [1, 2]. Globally, it is estimated that one-third of the population is infected with intestinal parasites most of which are found in developing countries [3]. Out of 3.5 billion people infected with intestinal parasites globally, about 450 million had clinical symptoms. More than 2 billion people are chronically infected with intestinal parasites. Above all, more than 200,000 deaths occur due to intestinal parasite infections every year. The global disease burden report showed that approximately 800 million people had Trichuriasis, and one billion had Ascariasis and Hookworm infections [4–6].

Food handlers play a major role in ensuring food safety in processing, storage, and preparation chain. A food handler is anyone who works in eating and drink establishments and handles food or contacts with any equipment or utensils that are likely to be in contact with food, such as cutlery, plates, bowls, or chopping boards [7–9]. Parasitic infections which belong to neglected tropical diseases are caused by various species of helminthes and protozoan. Parasitic diseases remain one of the most common types of human infection among food handlers throughout the world and are still causes of human morbidity and mortality. Food handlers infected with intestinal parasites are potential sources of infections for customers [10, 11].

In Ethiopia the prevalence of intestinal parasite infection among food handlers ranges from 14.5 to 44.1% out of which Giardia lamblia, *Entamoeba histolytica*, *schistosomiasis*, *Hookworm*, and *Ascariasis* infections were the most frequent [4, 12, 13].

Despite their being potential source of infection little is known about the magnitude of intestinal parasite infection and the factors affecting it among food establishment workers in the study area. Therefore, this study aimed to determine the prevalence and associated factors of intestinal parasites among food handlers in eating and drinking establishments of Chagni district.

## Main text

### Methods

#### Study design and setting

An institution based cross-sectional study was conducted from March to April 2018, in the food and drink service establishments of Chagni Town, Northwest Ethiopia. The town is found in Awi zone, Amhara National Regional State. The town is located 52 km from the zonal capital and 505 km from Addis Ababa the capital of Ethiopia. There were 255 legally registered food and drink serving establishments. These establishments include 23 hotels, 42 restaurants, 172 cafeterias, and 18 butcher shops with 127 food handlers working in hotels, 147 in restaurants, 430 in cafeterias, and 63 in butcher shops.

#### Sample size determination and sampling procedure

Study participants were selected by simple random sampling technique after proportional allocation of participants from all food and drink serving establishments. Seventy three from hotels, 85 from restaurants, 248 from cafeterias, and 36 from butcher shops were included. The minimum sample size 442 was calculated using the single proportion formula, considering the following assumption: 14.5% prevalence of intestinal parasites among food handlers in Aksum town [12], 95% level of confidence, 5% margin of error, and 10% non-response rate.

The dependent variable intestinal parasite infection was presence of one or more intestinal parasites on microscopic examinations of stool specimen. Independent variables were socio-demographic factors, regular medical checkup, work experience, food handlers’ hygiene and safety, food and drink establishments’ characteristics, and regulatory-body related issues.

#### Data collection tool and procedure

Data were collected using a structured interviewer administered questionnaire and laboratory diagnostic tests. Microscopic examination of stool specimen was done using saline wet mount which was prepared by mixing a small quantity (about 2 mg) of fresh stool with a drop of saline placed on a clean glass slide. The saline wet mount was used to examine presence of trophozoites and cysts of protozoa or eggs and larvae of helminthes. Two days’ intensive training was given to data collectors and supervisors on the objective of the study, the confidentiality of information, and techniques for conducting the interview. Two environmental health and four medical laboratory professionals participated in the data collection. Pretests were conducted on 5% of the food handlers who were not included in the study. Food handlers were asked to give fresh stool specimen using containers which are leak-proof, clean, dry, and free from traces of disinfectant and contamination with urine or feces. During each procedure in the stool diagnostic tests, two to three medical laboratory professionals reviewed for the ascertainment of cases of intestinal parasites. Finally, tests reviewed two times were reported as positive or negative for intestinal parasites.

#### Data processing and analysis

Data were entered into Epi-info version 3.5.3 and exported to SPSS version 20 for further analysis. Frequencies and percentages were computed for all variables. Data were presented in tables and graphs. The binary logistic regression statistical model was used to identify variables that were significantly associated with the outcome variable. Both Crude Odds Ratio (COR) and Adjusted Odds Ratio (AOR) with 95% confidence intervals were estimated to show the strength of associations. Variables with less than 0.2 P-values in the bi-variable analysis were entered into the multi-variable logistic analysis, while those with less than 0.05 P-values in the multivariable logistic regression analysis were considered as significantly associated with the prevalence of parasitic infection. The fitness of the model was checked by using the Hosmer–Lemeshow goodness-of-fit test. The Hosmer–Lemeshow test of significance in the multivariable analysis was 0.89.

### Results

#### Socio-demographic characteristics of food handlers

A total of 400 food handlers with a response rate of 90% were included. Out of the total food handlers, 294 (73.5%) were females and 106 (26.5%) were males. The mean age of participants was 26 (± 6) years with the majority 133 (30%) being younger than 22 years old (Table 1).

**Table 1:** Socio-demographic characteristics of food handlers, Chagni Town, Northwest Ethiopia, 2018 (n = 400)

Variables	Frequency	Percent
Sex		
Male	106	26.50
Female	294	73.50
Age		
< 22 years	120	30.00
22–25 years	99	24.75
26–28 years	83	20.75
> 28 years	98	24.50
Marital status		
Single	228	57.0
Married	163	40.80
Others*	9	2.30
Ethnicity		
Amhara	151	37.75
Awi	246	61.50
Others**	3	0.75
Religion		
Orthodox	369	92.30
Muslim	31	7.80
Educational status		
Illiterate	41	10.30
Primary school	195	48.80
Secondary school and above	164	41.00
Responsibility		
Cooker	46	11.50
Waiter and/dish washer	142	35.50
Serving both	212	53.00
Work experience		
< 1 year	73	18.30
1–2 years	144	36.00
Above 2 years	183	45.80
Monthly salary (Ethiopian Birr)		
< 500	122	30.50
500–700	85	21.25
701–1000	146	36.50
> 1000	47	11.75

Others*—divorced and widowed, Others**—Oromo and Shinasha

#### Food handlers’ hygiene and safety related factors

Out of the total (n = 400) food handlers, the majority 255 (63.8%) used uniforms/gown at work, 226 (56.5%) washed their hands regularly before handling and preparing food, 352 (88%) washed regularly before meals, 48 (12%) washed regularly after touching any body parts, 184 (46%) washed regularly after touching and handling any dirty material, and 360 (90%) did same after visiting toilets. Again, 281 (70.3%) trimmed finger nails, 73 (18.3%) reported to have medical check-ups for IP every 3 months, 88 (22%) checked in 6 months, and 239 (59.7%) every 9 month or above.

#### Food and drink establishment related factors

Of the total (n = 400) food handlers, 70 (17.5%) worked in hotels, 85 (21.3%) in restaurants, 211 (52.7%) in cafeterias and 34 (8.5%) in butcher’s. A total of 304 (76%) eating and drinking establishments had designated hand washing facilities, 156 (39%) flush type toilets, 212 (53%) dry type, and 32 (8%) have no toilet. One hundred sixty four (41%) of the food and drink establishments were inspected every 3 months for food hygiene and safety (Table 2).

**Table 2:** Hygiene and safety, regulatory related characteristics among food and drinking establishments in Chagni Town, Northwest Ethiopia, 2018

Variables	Diagnosed for at least one of the intestinal parasites	
	Yes Frequency (%)	No Frequency (%)
Type of establishment		
Hotels	11 (15.71)	59 (84.29)
Restaurants	11 (12.94)	74 (87.06)
Cafeterias	34 (16.11)	177 (83.89)
Butcher shops	3 (8.82)	31 (91.18)
Uniform/gown usage		
Yes	33 (12.94)	222 (87.06)
No	26 (17.93)	119 (82.07)
Regular hand washing before handling and preparing food		
Yes	36 (15.93)	190 (84.07)
No	23 (13.22)	151 (86.78)
Regular hand washing before meal		
Yes	46 (13.07)	306 (86.93)
No	13 (27.08)	35 (72.92)
Regular hand washing after touching any body parts		
Yes	3 (6.25)	45 (93.75)
No	56 (15.91)	296 (84.09)
Regular hand washing after touching and handling any dirty material		
Yes	21 (11.41)	163 (88.59)
No	38 (17.59)	178 (82.41)
Regular hand washing after visiting toilet		
Yes	47 (13.06)	313 (86.94)
No	12 (30.00)	28 (70.00)
Frequency of having shower per week		
More than 2 times per week	56 (14.62)	327 (85.38)
Once per week	3 (17.65)	14 (82.35)
Having trimmed fingernails		
Yes	34 (12.10)	247 (87.90)
No	25 (21.01)	94 (78.99)
Frequency of having medical checkup for IP		
Every 3 months	4 (5.48)	69 (94.52)
Every 6 months	12 (13.64)	76 (86.36)
Every 9 months and above	43 (18.00)	196 (82.02)
Availability of hand washing facility		
Yes	40 (13.16)	264 (86.84)
No	19 (19.79)	77 (80.21)
Liquid waste management options		
Closed type collection ditch	13 (15.66)	70 (84.34)
Open trench/pit	32 (13.91)	198 (86.06)
Open draining	14 (16.10)	73 (83.91)
Access and type of toilet facility		
Flush type	22 (14.10)	134 (85.90)
Dry type	34 (16.04)	178 (83.96)
No latrine	3 (9.40)	29 (90.63)
Regulatory inspections		
Every 3 months	24 (14.63)	140 (85.37)
Every 6 months	14 (13.73)	88 (86.27)
Every 9 months	2 (12.5)	14 (87.50)
Annually	19 (16.10)	99 (83.90)
Food hygiene and safety trainings		
Every 3 months	10 (10.64)	84 (89.36)
Every 6 months	21 (15.91)	111 (84.09)
Every 9 months	2 (9.5)	19 (90.48)
Annually	26 (16.99)	127 (83.01)

#### Prevalence of intestinal parasites among food handlers

From the total 400 participants 59 of them were infected with intestinal parasites making the prevalence 14.75% (95% CI 11.5%, 18%). The identified parasites were *Entamoeba histolytica* 34 (57.63%), *Ascaris lumbricoides* 11 (18.64%), Giardia lamblia 6 (10.17%), *Hookworm* 5 (8.47%), *Taenia species* 2 (3.39%) and *Hymenolepis nana* 1 (1.7%).

#### Factors associated with intestinal parasitosis among food handlers

In the multivariable logistic regression regular hand washing before meals and after visiting toilets, trimmed fingernails’ and frequent medical checkups for IP were found to be significantly associated with intestinal parasite infections.

The odds of being infected by intestinal parasites among food handlers who do not have regular hand washing before meal and after toilet was 4.77 (AOR 4.77; 95% CI 2.09, 10.87) and 3.39 (AOR 3.39; 95% CI 1.52, 7.57) times higher than their counter parts respectively. Food handlers with non-trimmed finger nails had 2.39 (AOR = 2.39; 95% CI 1.29, 4.42) times higher risk of intestinal parasitosis as compared to those with trimmed finger nails. When compared to participants who had regular medical checkup every 3 month the odds of having intestinal parasite infection was 3.54 (AOR = 3.54; 95% CI 1.11, 11.31) times higher among participants having checkup every 9 month and above (Table 3).

**Table 3:** Factors associated with prevalence of intestinal parasite among food handlers in food and drinking establishments in Chagni Town, Northwest Ethiopia, 2018

Variables	Parasitic infection		COR (95% CI)	AOR (95% CI)
	Yes Frequency (%)	No Frequency (%)		
Uniform/gown use				
Yes	33 (12.94)	222 (87.06)	1	1
No	26 (17.93)	119 (82.07)	1.47 (0.84, 2.57)	0.80 (0.37, 1.73)
Presence of hand washing facility				
Yes	40 (13.16)	264 (86.84)	1	1
No	19 (19.79)	77 (80.21)	1.63 (0.89, 2.97)	1.95 (0.82, 4.65)
Hand washing before meal				
Yes	46 (13.07)	306 (86.93)	1	1
No	13 (27.08)	35 (72.92)	2.47 (1.22, 5.02)	4.77 (2.09, 10.87)*
Hand washing after touching any body parts				
Yes	3 (6.25)	45 (93.75)	1	1
No	56 (15.91)	296 (84.09)	2.84 (0.85, 9.45)	3.22 (0.91, 11.37)
Hand washing after touching and handling any dirty material				
Yes	21 (11.41)	163 (88.59)	1	1
No	38 (17.59)	178 (82.41)	1.66 (0.93, 2.94)	1.69 (0.89, 3.21)
Hand washing after visiting a toilet				
Yes	47 (13.06)	313 (86.94)	1	1
No	12 (30.00)	28 (70.00)	2.85 (1.36, 5.99)	3.39 (1.52, 7.57)*
Having trimmed fingernails				
Yes	34 (12.10)	247 (87.90)	1	1
No	25 (21.01)	94 (78.99)	1.93 (1.09, 3.41)	2.39 (1.29, 4.42)*
Frequency medical Checkup for IP				
Every 3 months	4 (5.48)	69 (73.00)	1	1
Every 6 months	12 (13.64)	76 (86.36)	2.72 (0.84, 8.84)	2.89 (0.86, 9.69)
9 months and above	43 (18.00)	196 (82.01)	3.78 (1.31, 10.93)	3.54 (1.11, 11.31)*

* P value < 0.05

### Discussion

The overall prevalence of intestinal parasite infection in this study (14.8%) appeared to be consistent with the findings in Aksum Town, Northern Ethiopia 14.5% [12], Kenya, 14.4% [14], Bagalkot city, Karnataka, India 14.7% [15], and Sari, northern Iran, 15.5% [16]. But it was lower than a study done in Gambia [17], Ghana [18], Nigeria [19] and Sudan [20]. The possible reason might be the relative improvements made in Ethiopia in public health areas which include food hygiene and safety regulatory systems, food hygiene and safety trainings, medical checkup sessions in particular, and customer awareness and pressure in general [21–23]. It could also be due to the difference among studies in the method of assessing presence of parasites.

Regular hand washing at critical times like before meals and after visiting toilets had a statistically significant association with intestinal parasitosis. The odds of intestinal parasitosis among food handlers who didn’t practice regular hand washing before meal was higher than their counter parts. This finding appeared to be consistent with those of other studies which noted statistically significant results for the same predictor variables. These studies include Yebu town, southwest Ethiopia [13], Nairobi, Kenya [14], and Sari, northern Iran [16]. This is because hand washing before meal reduces intestinal parasite infection by preventing ingesting of the infective stage of the parasite by rates of 68% [24, 25].

The odds of intestinal parasitosis among food handlers who didn’t practice regular hand washing after visiting toilets were higher than those who did. This finding also appeared to be consistent with the findings of other studies conducted at Jimma University hospital, Ethiopia [26] and Nairobi, Kenya [14]. This mainly is due to the fact that hand washing reduces the risk of feco-oral transmission of these parasites [25].

Having trimmed fingernails was another factor that was significantly associated with the outcome of interest. The odds of intestinal parasitosis among food handlers who didn’t have trimmed fingernails were higher than their counter parts. This finding is consistent with studies conducted in Yebu town [13] Arba Minch [27], and Jimma [26]. The possible reason could be untrimmed fingernails hide disease causing infectious agent and also serve as a good mode of transmission. Weekly nail clipping was also found to decrease intestinal parasite re-infection rates by 49% [24, 28].

The odds of intestinal parasitosis among food handlers who did medical checkups every 9 month or above were higher than those who did every 3 month. This finding is supported by another study conducted in Aksum Town, Northern Ethiopia [12]. This could be because those who had frequent medical checkup sessions are more likely to receive anti-parasitic medications and health education than those who do not [29].

### Conclusions

According to the results of this study, the overall prevalence of intestinal parasite infection among food handlers was high. Regular hand washing practices at critical times, before meals and after visiting toilets, having trimmed fingernails, and frequent medical check-ups for intestinal parasites were statistically significant predictors for intestinal parasitosis among food handlers. Regulatory standards that enforce prior medical checkup and certification of food handlers should be advocated, practiced, monitored, and evaluated as per the standard at all levels. Hygiene promotion for food handlers, especially hand washing at critical times, including nail clipping should be practiced. Food handlers shall also clip their finger nails regularly.

## Limitations

Since it is a cross-sectional study temporal relationship cannot be established. The method of identifying intestinal parasites that is the conventional saline wet mount has lower sensitivity, compared to concentration methods. This may underestimate the prevalence of intestinal parasite infection in the study.

## Data Availability

Data will be available upon a reasonable request made to the correspondent author.

## Abbreviations

AOR: Adjusted Odds Ratio
CI: confidence interval
COR: Crude Odds Ratio
IP: intestinal parasite
mg: milligram
WHO: World Health Organization
